# Supplementary material for: Protective Role of Galanin during Chemically Induced Inflammation in Zebrafish Larvae
Source: Biology (Basel). 2021 Jan 30;10(2):99. doi: 10.3390/biology10020099 (PMC7911020; doi:10.3390/biology10020099)
Supplement: Supplementary file 1 [file biology-10-00099-s001.pdf]

Supplementary figure 1

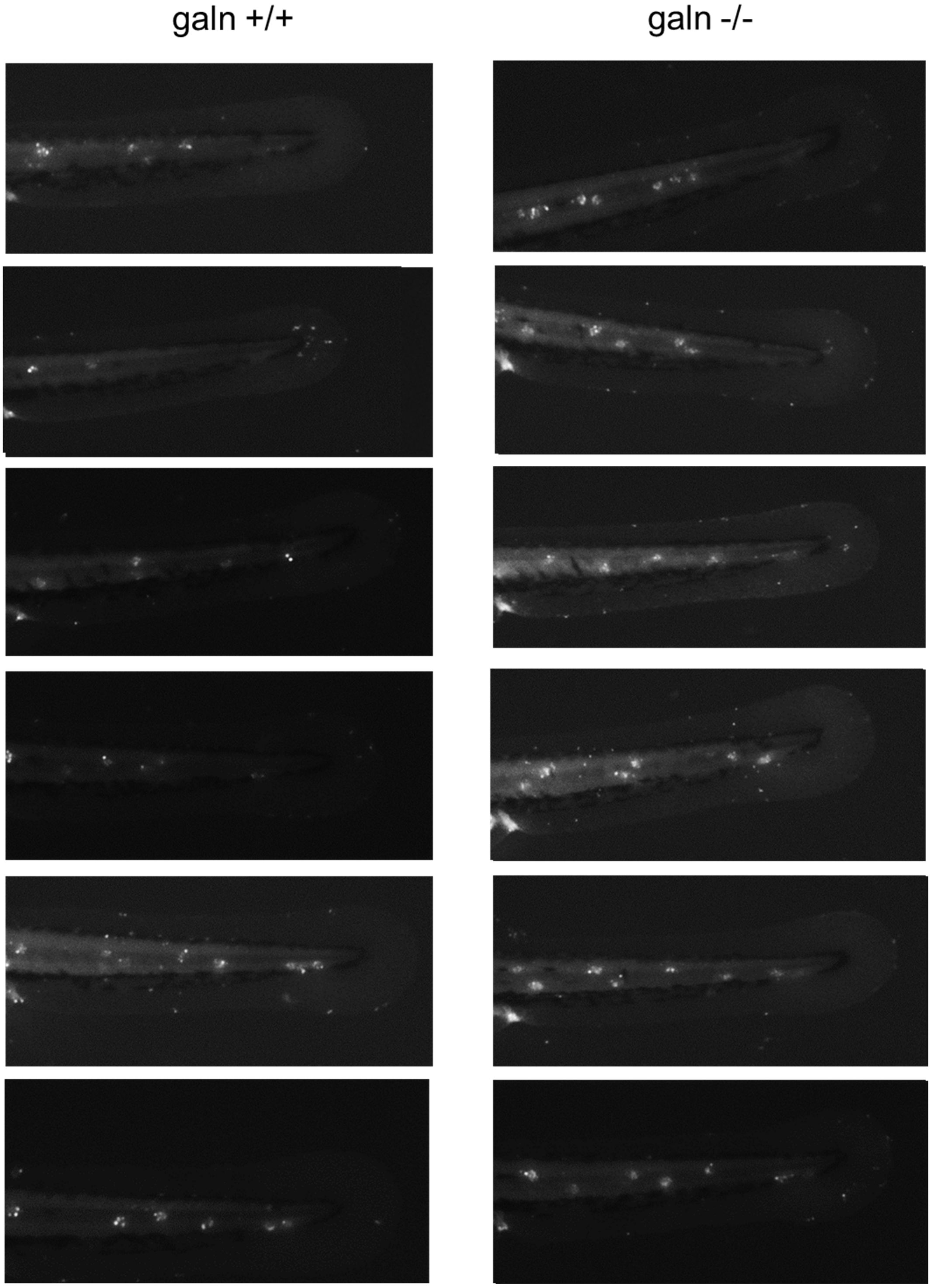

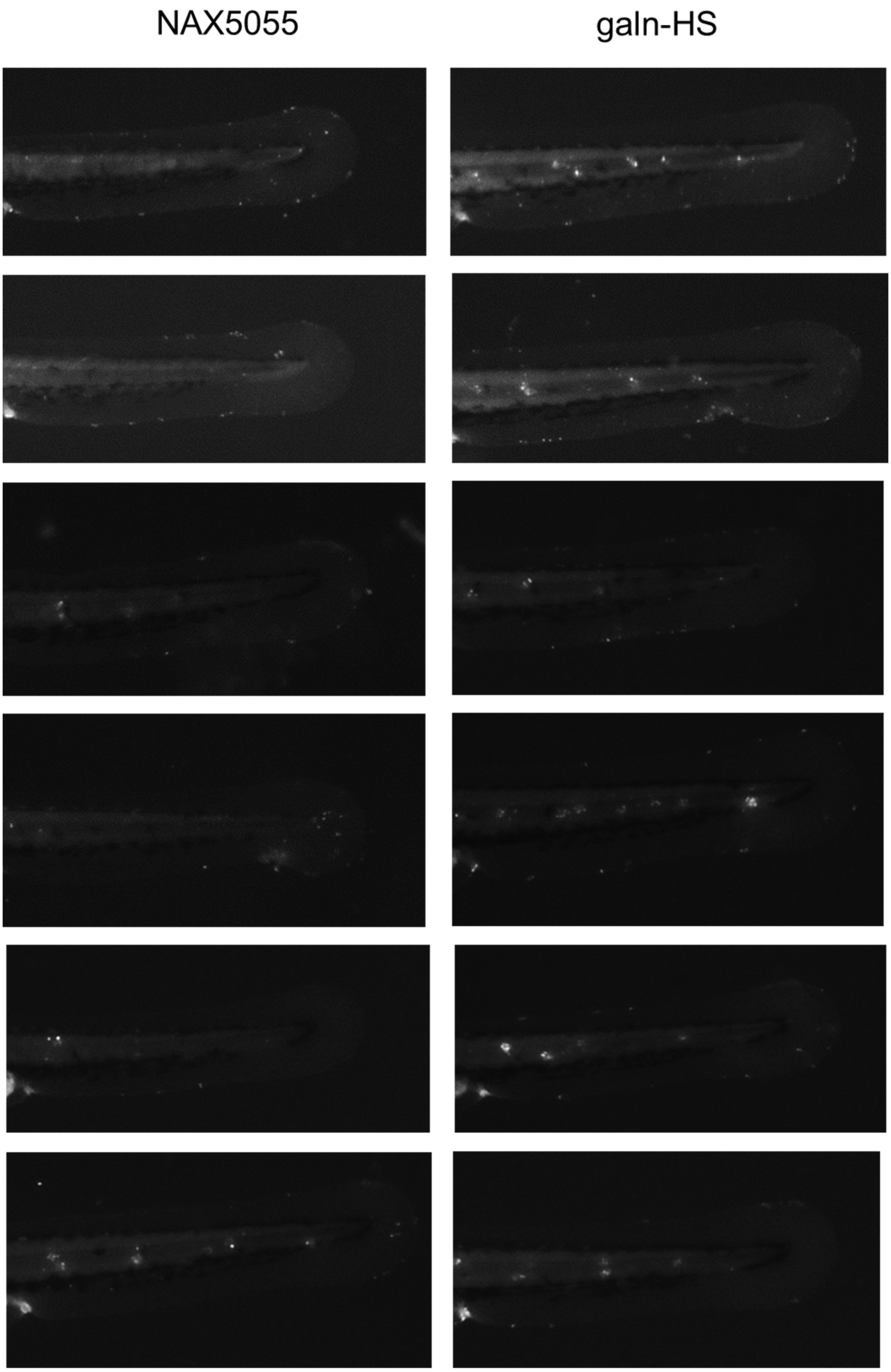

Figure **S1. Cell degeneration after copper sulfate treatment with acridine orange staining.** Neuromasts degeneration after copper sulfate exposure in the wild-type *galn*$^{+/+}$ group, a line with galanin knockout *galn*$^{-/-}$, treated with galanin analogue NAX5055 and a line with galanin overexpression Tg(*hsp70l:galn*).

Supplementary figure 2

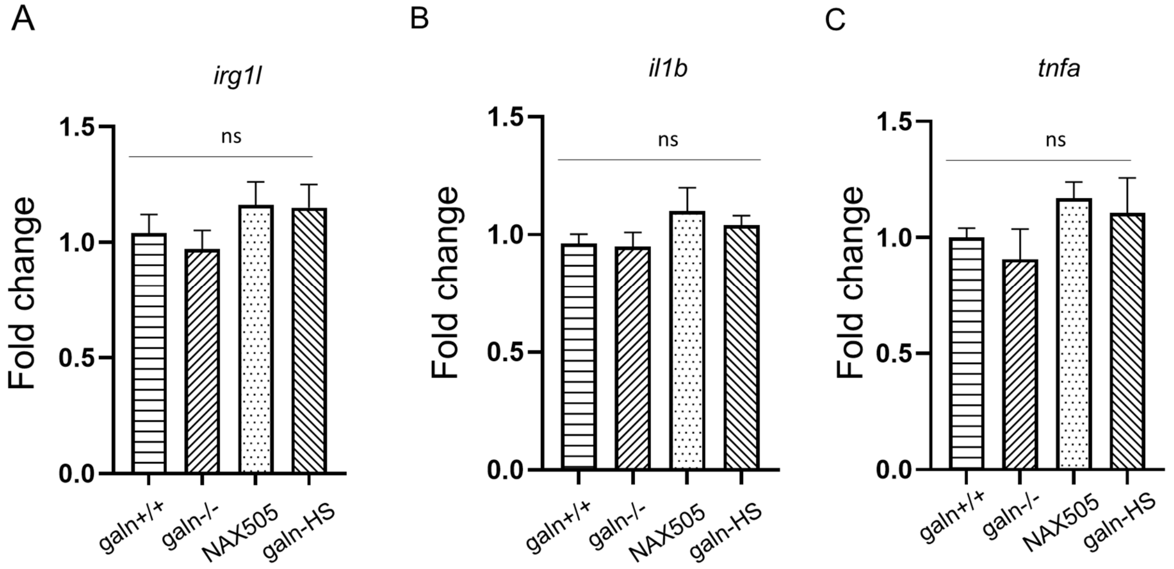

Figure **S2. quantitative PCR of basal expression of *irg1l, il1b* and *tnfa*.** (**A**–**C**) qPCR of *irg1l, il1b* and *tnfa* after vehicle incubation with PBS was performed at 3dpf. Data, mean±s.e.m. were pulled from three independent experiments. (determined using ANOVA with Tukey's post hoc test); ns, not-significant.
